# Transcription of NOD1 and NOD2 and their interaction with CARD9 and RIPK2 in IFN signaling in a perciform fish, the Chinese perch, *Siniperca chuatsi*

**DOI:** 10.3389/fimmu.2024.1374368

**Published:** 2024-04-23

**Authors:** Xue Yun Peng, Kai Lun Wang, Li Li, Bo Li, Xiang Yang Wu, Zhi Wei Zhang, Nan Li, Lan Hao Liu, P. Nie, Shan Nan Chen

**Affiliations:** ^1^ State Key Laboratory of Freshwater Ecology and Biotechnology, and Key Laboratory of Aquaculture Disease Control, Institute of Hydrobiology, Chinese Academy of Sciences, Wuhan, Hubei, China; ^2^ College of Advanced Agricultural Sciences, University of Chinese Academy of Sciences, Beijing, China; ^3^ School of Marine Science and Engineering, Qingdao Agricultural University, Qingdao, Shandong, China; ^4^ Laboratory for Marine Biology and Biotechnology, Qingdao Marine Science and Technology Center, Qingdao, Shandong, China

**Keywords:** NOD1, NOD2, CARD9 and RIPK2, ISG, IFN, *Siniperca chuatsi*

## Abstract

NOD1 and NOD2 as two representative members of nucleotide-binding oligomerization domain (NOD)-like receptor (NLR) family play important roles in antimicrobial immunity. However, transcription mechanism of *nod1* and *nod2* and their signal circle are less understood in teleost fish. In this study, with the cloning of *card9* and *ripk2* in Chinese perch, the interaction between NOD1, NOD2, and CARD9 and RIPK2 were revealed through coimmunoprecipitation and immunofluorescence assays. The overexpression of NOD1, NOD2, RIPK2 and CARD9 induced significantly the promoter activity of NF-κB, IFNh and IFNc. Furthermore, it was found that *nod1* and *nod2* were induced by poly(I:C), type I IFNs, RLR and even NOD1/NOD2 themselves through the ISRE site of their proximal promoters. It is thus indicated that *nod1* and *nod2* can be classified also as ISGs due to the presence of ISRE in their proximal promoter, and their expression can be mechanistically controlled through PRR pathway as well as through IFN signaling in antiviral immune response.

## 1 Introduction

Pathogenic infections can activate the host immune defense system, which is initiated by the recognition of pathogenic components, called pathogen-associated molecular patterns (PAMPs), by some families of pattern recognition receptors (PRRs), including Toll-like receptors (TLRs), retinoic acid-inducible gene I (RIG-I)-like receptors (RLRs), nucleotide-binding oligomerization domain (NOD)-like receptors (NLRs), C-type lectin receptors (CLRs) and intracellular DNA sensors (1). The activated PRRs recruit adaptors and trigger downstream signaling pathways, which promote the expression of immune-related cytokines, such as interferons (IFNs) and interleukin (IL)-1β (1). IFNs are class II cytokines and play important roles in antiviral immunity (2). To date, vertebrate IFNs are identified into four types (I, II, III and IV), which use different receptors to activate Janus kinase-signal transducer and activator of transcription (JAK-STAT) signaling for the induction of IFN-stimulated gene (ISGs) through IFN-stimulated response elements (ISREs) (3–6).

RLRs are the critical virus-RNA recognition receptors in cytoplasm. Almost all vertebrate RLR family has three members, including RIG-I (also known as DEAD box polypeptide 58, DDX58), melanoma differentiation-associated gene 5 (MDA5, also called IFN induced with helicase C domain 1, IFIH1) and laboratory of genetics and physiology 2 (LGP2, or DExH box polypeptide 58, DHX58), although the functional *rig-I* gene may have been lost in some taxa of vertebrates, such as chicken (*Gallus gallus*), Chinese tree shrew (*Tupaia belangeri chinensis*) and teleost fish in the Acanthomorphata (7). Activated RIG-I and MDA5 recruit mitochondrial antiviral signaling protein (MAVS), leading to the phosphorylation of critical kinases, such as TANK binding kinase 1 (TBK1) and some transcription factors, including IFN regulatory factor 3 (IRF3) and IRF7, which bind to the positive regulatory domains (PRDs) or ISRE in promoter region to upregulate the expression of IFNs and ISGs and inhibit virus proliferation (1).

NOD1 and NOD2 are the two representative members of NLR family and possess functional recognition to the bacterial γ-D-glutamyl-meso-diaminopimelic acid (iE-DAP) and muramyl dipeptide (MDP), respectively (8). By contrast, NOD1 and NOD2 have similar domain organization with caspase activation recruitment domain (CARD) at N-terminal region, followed by a NAIP, CIITA, HET-E and TP1 (NACHT) domain and a leucine-rich repeat (LRR) domain at C-terminus, although NOD2 has another CARD than NOD1 (9). The CARD of NODs is a key structure to interact with downstream adaptor, receptor-interacting serine-threonine kinase 2 (RIPK2, also known as RIP2) to activate NF-κB signaling and promote inflammatory response (9, 10). Interestingly, the CARD9, a critical adaptor protein, is associated with NOD2 to trigger p38 MAPK and JNK signaling, but not NF-κB (11). In fact, CARD9 is involved in CLR-mediated activation of NF-κB pathway (11). Moreover, it is known that NOD1/NOD2 and RIPK2 complex can induce type I IFN through TRAF3-TBK1-IRF3/7 cascade (9).

In teleost fish, RIG-I/MDA5-MAVS-IRF3/7 and NOD1/NOD2-RIPK2-NF-κB pathways have been identified respectively in some species, such as zebrafish (*Danio rerio*), grass carp (*Ctenopharyngodon idella*), rainbow trout (*Oncorhynchus mykiss*), common carp (*Cyprinus carpio*), black carp (*Mylopharyngodon piceus*), orange-spotted grouper (*Epinephelus coioides*) and crucian carp (*Carassius auratus*) (12–19). Importantly, fish RLR- and NLR-mediated signaling in antiviral immunity has been confirmed to possess crosstalk with NOD1 acting as a positive regulator of MDA5-MAVS pathway (20). In fact, current research on the function of NOD1 and NOD2 in fish is mainly concerned with the ligand recognition and downstream signaling regulation, such as the transcription of type I IFNs (4). However, the knowledge concerning NOD1/NOD2 signaling circle is rather limited, especially in transcriptional regulation of these two genes.

Chinese perch (*Siniperca chuatsi*), belonging to the order Perciformes, has high economic value in Chinese freshwater aquaculture, and understanding of its immune system has attracted attention of aquaculture scientists in order to develop effective preventive and control measures against diseases (21–23). In this study, the adaptors of NODs, CARD9 and RIPK2, were cloned in Chinese perch, and the interactive relationship between CARD9/RIPK2 and NOD1/NOD2 was verified through coimmunoprecipitation and cellular colocalization, respectively. Moreover, poly(I:C) induced expression of *nod1* and *nod2* was examined, and the promoter sequence of *nod1* and *nod2* genes was bioinformatically analyzed with the possible identification of ISRE. Then, luciferase reporter assay was performed to examine if IFNs in Chinese perch could induce the promoter activity of *nod1* and *nod2*, and *vice versa* if NOD1, NOD2, RIPK2 and CARD9 could induce the promoter activity of IFNs and NF-κB. These results indicated that NOD1 and NOD2 should be involved in antiviral immune response in an IFN-dependent manner at least in lower vertebrates.

## 2 Materials and methods

### 2.1 Fish, cell lines and virus

Chinese perch were obtained from a fish farm in Hubei Province, China, and maintained in aerated tanks at 28 °C with recirculating fresh water for two weeks prior to the experiments. All animal experiments were conducted in accordance with the Guiding Principles for the Care and Use of Laboratory Animals and were approved by the Institute of Hydrobiology, Chinese Academy of Sciences.

The mandarin fish fry cell line (MFF-1) and human embryonic kidney 293T (HEK293T) cells were maintained in Dulbecco’s modified Eagle’s medium (DMEM; Gibco) with 10% fetal bovine serum (FBS; Gibco) and 5% CO_2_ at 28°C and 37°C, respectively. The infectious hemorrhagic syndrome virus (IHSV) was propagated in MFF-1 cells, and viral titers were determined according to a previous report (24).

### 2.2 RNA extraction, cDNA synthesis and gene cloning

DNase I reagent (Thermo Scientific) was used at 37°C to remove genomic DNA remnants in total RNA samples, which were extracted from fish tissues or cells using TRIzol^®^ reagent (Ambion) following the manufacturer’s protocol. Next, the RevertAid™ First Strand cDNA Synthesis Kit (Thermo Scientific) was used to synthesize first strand cDNA according to the manufacturer’s instruction. Full-length open reading frames (ORFs) of *ripk2*, *card9*, *nod1* and *nod2* were generated through PCR amplification by using Phanta Max Master Mix high-fidelity DNA polymerase (Vazyme) with gene-specific primers (referring to **Supplementary Table 1**) which were designed based on the predicted sequences (XM_044218208.1 and XM_044195794.1 from NCBI database) and the reported sequences (KY974318 and KY974317) (25). All PCR products were confirmed by sequencing.

### 2.3 Sequence analyses

The online Translate tool (http://web.expasy.org/translate/) was used to predict amino acid sequences. The conserved domain of proteins and transmembrane region was determined by BLASTP program based on Conserved Domains Database (CDD) and by TMHMM Server v.2.0 program (http://www.cbs.dtu.dk/services/TMHMM-2.0/), respectively. The AnimalTFDB v4.0 software (http://bioinfo.life.hust.edu.cn/AnimalTFDB4/) was used to predict the ISRE site of *nod1* and *nod2* genes. Multiple alignments of protein sequences were performed by using the Clustal X and GeneDoc program. Phylogenetic trees were generated by using neighbor joining (NJ) method with 1000-time bootstrap in MEGA-X package. For gene collinearity analysis, data were collected from the available assemblies, including *Homo sapiens* (human, version: GRCh38.p14, NCBI accession: GCF_000001405.40), *Mus musculus* (house mouse, version: GRCm39, GCF_000001635.27), *Gallus gallus* (chicken, version: bGalGal1.mat.broiler.GRCg7b, GCF_016699485.2), *Danio rerio* (zebrafish, version: GRCz11, GCF_000002035.6) and *Siniperca chuatsi* (version: ASM2008510v1, GCF_020085105.1).

### 2.4 Construction of plasmids and cell transfection

The IFN expression plasmids (pcDNA3.1-IFNh, pcDNA3.1-IFNc and pcDNA3.1-IFN-γ) and promoter luciferase reporter plasmids, including pGL3-NF-κB-pro-Luc, pGL3-IFNh-pro-Luc (HP1) and pGL3-IFNc-pro-Luc (CP1), were described in previous reports (26, 27). The pcDNA3.1/Myc-His(-) and p3XFLAG-CMV-14 vectors were used to construct eukaryotic expression plasmids in this study. The full-length ORF fragments were generated by PCR procedure with the protocol as the follows: initial denaturation for 3 min at 95°C, 35 cycles for 30 s at 95°C, 30 s at 55°C and 2 min at 72°C, followed by a further 10 min at 72°C. The specific primers used in the amplification are shown in **Supplementary Table 1**. The ORFs of *nod1* and *nod2* were inserted into the vector to construct pcDNA3.1-NOD1-Myc and pcDNA3.1-NOD2-Myc through *Eco*RI/*Kpn*I site, respectively. The ORFs of *card9* and *ripk2* were cloned into the *Not*I/*Xba*I site of p3XFLAG-CMV-14 to obtain p3XFLAG-CARD9-FLAG and p3XFLAG-RIPK2-FLAG, respectively. The *card9*, *ripk2* and *mda5* with an HA-tag-encoding sequences (5’-*tacccatacgacgtcccagactacgct*-3’) were ligated into the *Eco*RI/*Xba*I site of p3XFLAG, *Eco*RI/*Kpn*I site of pcDNA3.1, and *Not*I/*Kpn*I site of pcDNA3.1 to generate p3XFLAG-CARD9-HA, p3XFLAG-RIPK2-HA and p3XFLAG-MDA5-HA, respectively. The plasmids were extracted and purified by using E.Z.N.A.^®^ Endo Free Plasmid Mini Kit II (Omega Bio-tek) following the manufacturer’s instruction. Cell transfection was performed according to the previous reports (26, 28), and the used transfection reagents for HEK293T and MFF-1 cells were Lipofectamine 2000 (Invitrogen) and FuGENE^®^ HD Transfection Reagent (Promega), respectively.

### 2.5 Coimmunoprecipitation

HEK293T cells (about 5 × 10^6^) were seeded into 90 mm cell culture dishes overnight and were co-transfected with 4 µg of each plasmid according to a series of combinations, including pcDNA3.1-NOD1-Myc + p3XFLAG-CMV-14 empty vector, pcDNA3.1-NOD1-Myc + p3XFLAG-CARD9-FLAG, pcDNA3.1-NOD1-Myc + p3XFLAG-RIPK2-HA; pcDNA3.1-NOD2-Myc + p3XFLAG-CMV-14 empty vector, pcDNA3.1-NOD2-Myc + p3XFLAG-CARD9-HA, pcDNA3.1-NOD2-Myc + p3XFLAG-RIPK2-HA; p3XFLAG-RIPK2-FLAG + p3XFLAG-CMV-14 empty vector, and p3XFLAG-RIPK2-FLAG + p3XFLAG-CARD9-HA. 48 hours after transfection, the supernatant medium was removed and the cells were washed with phosphate buffered saline (PBS, pH 7.0). Each sample of cells was added with 400 µL lysis buffer (Thermo Fisher Scientific) containing 4 µL protease inhibitor mixture (Thermo Fisher Scientific), and was lysed on the rotational mixer at 4 °C for 24 hours. Next, cell fragments were removed by centrifugation at 2000 × *g* for 10 min, and the supernatants were incubated by overnight rotating with anti-FLAG or anti-HA Immunomagnetic Beads (Dia-An Biotech) at 4°C. After wash for five times with PBS, protein samples were collected from beads through boiling.

Western blotting assay was performed as described in a previous study (3, 27). The primary antibodies included mouse monoclonal antibody (mAb; anti-FLAG M2, #F1804, Sigma-Aldrich) with the dilution ratio of 1:5000, mouse anti-Myc-Tag mAb (#2276, Cell Signaling Technology, 1:5000), rabbit anti-HA-Tag mAb (#3724, Cell Signaling Technology, 1:5000), and the secondary antibodies included HRP-conjugated mouse anti-rabbit IgG (#D110065-0100, Sangon Biotech, 1:5000) and HRP-conjugated goat anti-mouse IgG (#D110087-0100, Sangon Biotech, 1:5000).

### 2.6 Immunofluorescence assay

MFF-1 cells were seeded onto coverslips in 24-well plates and were co-transfected with 0.25 µg of each plasmid in same combinations as described above. 24 hours after transfection, the supernatants were removed, and the cells were washed with PBS and fixed by using 4% paraformaldehyde at 28°C for 40 min. Next, the cells were washed four times with PBS and incubated with 0.2% Triton X-100 to permeate the cell membrane at 28°C for 12 min. After being washed with PBS for four times, the cells were blocked by 5% BSA at 37°C for 1 hour and incubated with the tag-antibodies described above and the fluorescein-labelled secondary antibodies, including Goat anti-Mouse IgG (H+L) Cross-Adsorbed Secondary Antibody, Alexa Fluor™ 594 (Thermo Fisher Scientific) or Goat anti-Rabbit IgG (H+L) Cross-Adsorbed Secondary Antibody, Alexa Fluor™ 488 (Thermo Fisher Scientific). Subsequently, the coverslips with labelled cells were sticked on the microscope slides by using ProLong™ Diamond Antifade Mountant with DAPI (Thermo Fisher Scientific). The cells were observed under a Leica confocal microscope (40 × magnification, oil immersion lens).

### 2.7 Luciferase activity assays

MFF-1 cells were seeded in 48-well plates and were transfected with a series of plasmid combinations (empty/expression plasmids + luciferase reporter plasmids), including 0.1 μg pcDNA3.1/p3XFLAG-CMV-14, 0.1 μg pcDNA3.1-NOD1-Myc/pcDNA3.1-NOD2-Myc/p3XFLAG-RIPK2-FLAG/p3XFLAG-CARD9-HA/p3XFLAG-MDA5-HA, 0.1 μg pGL3-NF-κB-pro-Luc/pGL3-IFNh-pro-Luc (HP1)/pGL3-IFNc-pro-Luc (CP1)/pGL3-NOD1-pro-Luc (wt)/pGL3-NOD1-pro-Luc (mut)/pGL3-NOD2-pro-Luc (wt)/pGL3-NOD2-pro-Luc (mut) and 0.02 μg pRL-TK. 24 hours after transfection, the cells of each well were washed once with PBS and were lysed by adding 100 μL Passive Lysis Buffer (Promega) for half hour. The Luciferase^®^ Reporter Assay System (Promega) was used to determine the luciferase activity by running GloMax^®^-Multi JR detection system (Promega).

HEK293T cells were seeded into 6 well plates and were transfected with 1 μg pcDNA3.1 empty vector (control), pcDNA3.1-IFNh, pcDNA3.1-IFNc or pcDNA3.1-IFN-γ to generate recombinant protein of IFNh, IFNc or IFN-γ, respectively. The control group and recombinant proteins of IFNh/IFNc/IFN-γ in the cell precipitation and supernatant medium were detected by Western blotting with IFN-specific antibodies, respectively (27). Then, the control and supernatant medium with recombinant IFNs were diluted 10 times for incubation. MFF-1 cells were seeded into 48 well plates and were transfected with 0.01 μg pRL-TK and 0.1 μg pGL3-NOD1-pro-Luc (wt)/pGL3-NOD1-pro-Luc (mut)/pGL3-NOD2-pro-Luc (wt)/pGL3-NOD2-pro-Luc (mut), respectively. After transfection for 24 hours, the MFF-1 cells were incubated separately with control medium and recombinant protein of IFNh (final concentration of 107.5 ng/mL)/IFNc (266.67 ng/mL)/IFN-γ (57.92 ng/mL) for 24 hours to detect the luciferase activity.

### 2.8 Gene expression analyses

To investigate the tissue distribution of *nod1*, *nod2*, *card9* and *ripk2*, three healthy Chinese perch with a body length of about 10 cm were sacrificed to collect different organs/tissues, including fin, head-kidney, truck-kidney, spleen, skin, liver, gill, eye, and intestine, for qRT-PCR detection. To investigate whether the four genes (*nod1*, *nod2*, *card9* and *ripk2*) could be induced by poly(I:C), IFN-γ, IFNh and IFNc, MFF-1 cells (5.0 × 10^5^) were seeded in 12-well plates and were separately transfected with 50 μg (final concentration of 41.67 μg/mL) poly(I:C) by using 1 μL Lipofectamine 2000 transfection reagent (Thermo Fisher Scientific) or directly incubated with 100 μL prepared culture medium supernatant containing the recombinant protein of IFNγ/IFNh/IFNc, as described above. The samples were collected at 3, 6, 12 and 24 hours post-stimulation, respectively. For IHSV infection, MFF-1 cells (5.0 × 10^5^) were seeded in 12-well plates and were infected with IHSV (MOI = 0.01) or without virus (control group), and the samples were collected at 12, 24, 36 and 48 hours post-infection, respectively.

### 2.9 Quantitative real-time PCR

The cDNA synthesis from total RNA was performed with All-in-One RT SuperMix Perfect for qPCR kit (Vazyme). A total of 20 μL reaction system consisted of 10 μL PowerUP™ SYBR™ Green Master Mix (ABI), 2 μL primer mixture, 7 μL sterile water, and 1 μL cDNA template. The qRT-PCR was performed on the QuantStudiO™ 3 Real-Time PCR Instrument (96-Well Block, ABI) with the following protocol: initial denaturation for 2 min at 50°C, 2 min at 95°C, 50 cycles for 15 s at 95°C, 30 s at 58°C, followed by a further 15 s at 95°C, 1 min at 60°C, 1 s at 95°C. The β-actin was used as the internal reference gene to calculate the folding change of expression level of target genes using the 2^(-ΔΔCt)^ method. The specific primers are listed in **Supplementary Table 1**.

### 2.10 Statistical analyses

All experiments were repeated separately at least three times. Results were analyzed statistically with Student’s *t*-test in SPSS 16.0 software. Significant differences were indicated with * *P* < 0.05, and ** *P* < 0.01, respectively. The data were presented as mean ± standard error (SE).

## 3 Results

### 3.1 Identification of *ripk2* and *card9* in Chinese perch

**Figure 1:**
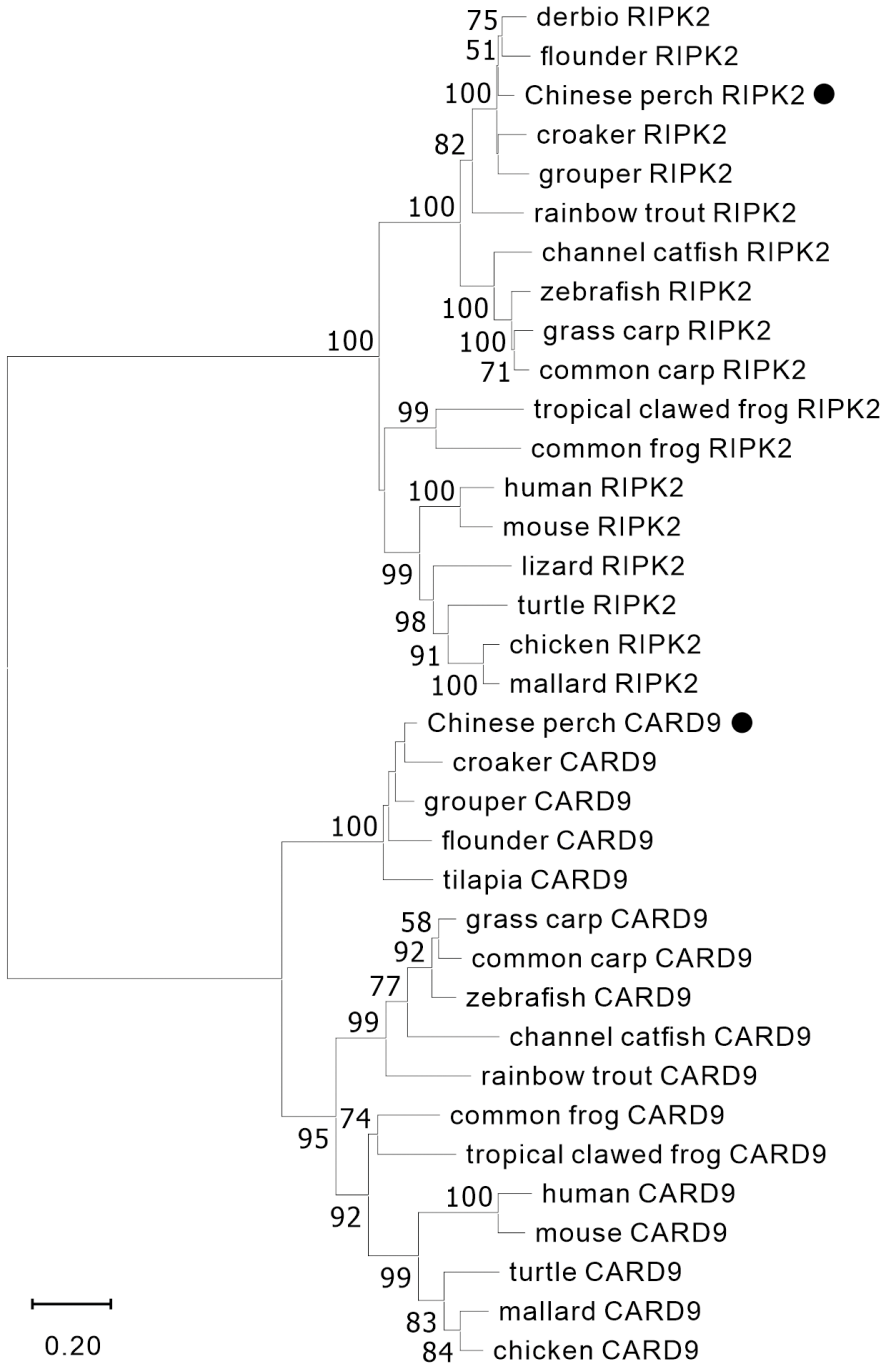
Phylogenetic analysis of vertebrate RIPK2 and CARD9. The phylogenetic tree was constructed by using MAGE-X software with neighbor-joining (NJ) method and bootstrapped 1000 times. The GenBank accession numbers of the protein sequences are as the followings, human_CARD9 (NP_434700.2), zebrafish_CARD9 (XP_009300061.1), common_carp_CARD9 (BAC00527.1), grass_carp_CARD9 (XP_051748621.1), channel_catfish_CARD9 (XP_017307713.1), rainbow_trout_CARD9 (XP_021461047.1), mouse_CARD9 (NP_001032836.1), chicken_CARD9 (XP_046757916.1), mallard_CARD9 (XP_027326556.2), turtle_CARD9 (XP_027679145.1), croaker_CARD9 (TMS01615.1), grouper_CARD9 (XP_033474752.1), flounder_CARD9 (XP_019964458.1), tilapia_CARD9 (XP_005464332.1), tropical_clawed_frog_CARD9 (NP_001120274.1), common_frog_CARD9 (XP_040180075.1), chicken_RIPK2 (NP_001026114.1), mallard_RIPK2 (XP_027308245.1), mouse_RIPK2 (NP_620402.1), zebrafish_RIPK2 (NP_919392.2), grass_carp_RIPK2 (AYN79345.1), common_carp_RIPK2 (XP_018955475.1), channel_catfish_RIPK2 (WBW48352.1), rainbow_trout_RIPK2 (AIZ74459.1), human_RIPK2 (NP_003812.1), lizard_RIPK2 (XP_008106622.1), turtle_RIPK2 (XP_007052976.3), derbio_RIPK2 (URH23960.1), flounder_RIPK2 (XP_019958344.1), croaker_RIPK2 (XP_010731625.1), grouper_RIPK2 (XP_033507423.1), common_frog_RIPK2 (XP_040210528.1), and tropical_clawed_frog_RIPK2 (XP_002939201.2).

**Figure 2:**
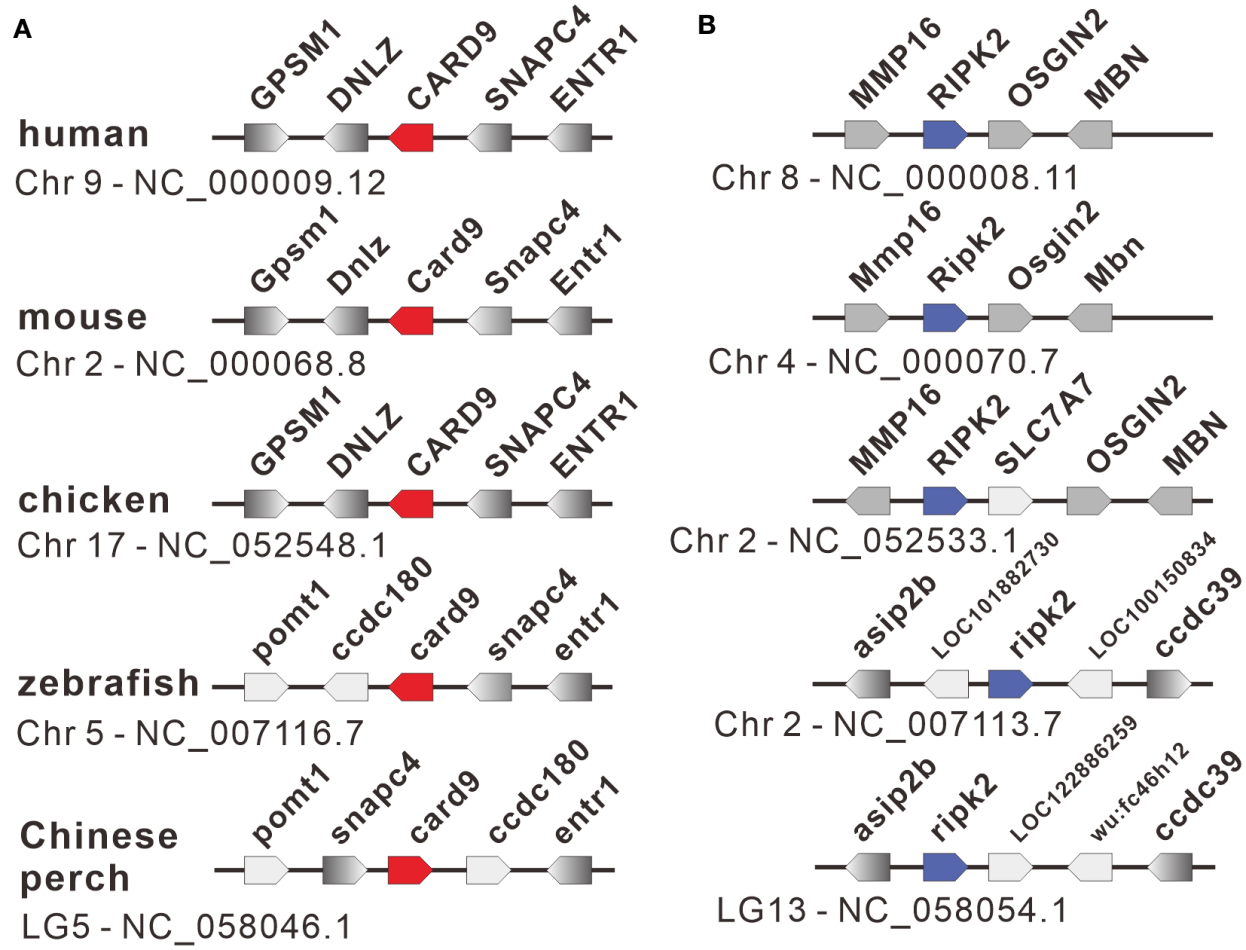
Gene synteny of *card9* **(A)** and *ripk2* **(B)** loci in vertebrates. The functional genes with transcription direction were indicated in arrow symbols, and the *card9*, *ripk2* and linked genes were dyed in red, blue, and gray, respectively.

The nucleotide sequences of two adaptors, RIPK2 and CARD9, were cloned from mixed cDNA samples of Chinese perch tissues and were deposited in the GenBank database with the accession numbers, OR713117 and OR713116, respectively. The full-length ORF of *ripk2* and *card9* contained 1806 and 1623 base pairs, respectively, which were predicted to encode 601 and 540 amino acids. The RIPK2 and CARD9 protein sequences shared medium identity with human (46.6%/35.6%), mouse (46.4%/37.6%), chicken (44.6%/35.8%) and zebrafish (69.8%/43.2%), respectively. The multiple alignments and domain prediction revealed that vertebrate RIPK2 has a conserved serine-threonine kinase domain (KD) at N-terminal region and a conserved C-terminal CARD domain, and CARD9 has a N-terminal CARD domain and a coiled-coil (CC) domain, while no transmembrane motif (TM) was found in the two molecules (**Supplementary Figures 1**, **2**). Phylogenetic analysis showed that RIPK2 and CARD9 were formed into a separate clade with their fish homologues, respectively (**Figure 1**). Moreover, in synteny analysis, vertebrate *card9* was found to be linked with conserved small nuclear RNA activating complex polypeptide 4 (*snapc4*) from fish to human, and tetrapod *ripk2* is located on a conserved genetic region which contains matrix metallopeptidase 16 (*mmp16*) and oxidative stress induced growth inhibitor family member 2 (*osgin2*) (**Figure 2A**). However, fish *ripk2* was sandwiched with agouti signaling protein, nonagouti homolog (mouse) 2b (*asip2b*) and coiled-coil domain containing 39 (*ccdc39*) (**Figure 2B**).

### 3.2 Expression of *nod1*, *nod2*, *ripk2* and *card9* genes

**Figure 3:**
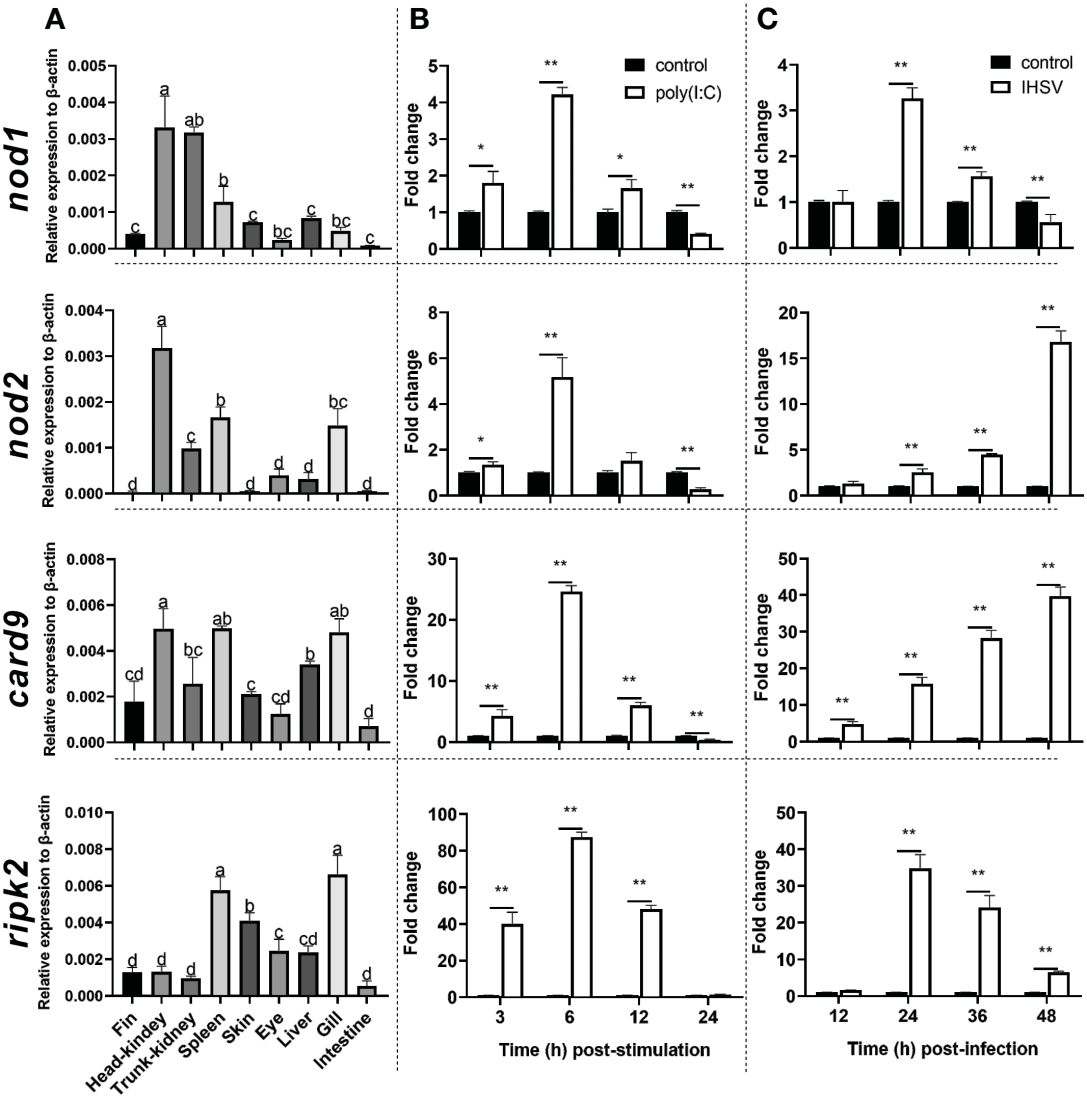
Tissue distribution **(A)** and induction by poly(I:C) **(B)** and IHSV **(C)** of *nod1*, *nod2*, *card9* and *ripk2* genes in Chinese perch. Chinese perch (n = 3) organs/tissues, including head-kidney, trunk-kidney, spleen, liver, intestine, fin, gill, eye, and skin, were collected for constitutive expression analyses of target genes. For inducible expression analyses, MFF-1 cells (5.0 × 10^5^) were transfected with a final concentration of 41.67 μg/mL poly(I:C) and were collected at 3, 6, 12 and 24 h post-stimulation, respectively. Untreated cells were set as control. For IHSV infection, MFF-1 cells (5.0 × 10^5^) were seeded in 12-well plates and were infected with IHSV (MOI = 0.01) or without infection (control group), and the samples were collected at 12, 24, 36 and 48 hours post-infection, respectively. Gene expression was determined by qRT-PCR and was normalized against β-actin. Data were expressed as mean ± SE, with * indicating *P* < 0.05, **P < 0.01. ANOVA with post hoc Tukey test was performed to reveal significant difference through different letters (a > b > c > d), the bars indicate mean ± SE.

The qRT-PCR was used to detect the expression level of *nod1*, *nod2*, *ripk2* and *card9* genes in Chinese perch organs/tissues, including head-kidney, trunk-kidney, spleen, liver, intestine, fin, gill, eye, and skin. As shown in **Figure 3A**, the four genes were constitutively expressed in all detected organs/tissues, and *card9* mRNA was mainly enriched in head-kidney, spleen, and gill, while *ripk2* gene was expressed with highest transcription level in spleen and gill. Interestingly, *nod1* and *nod2* genes showed high expression levels in head-kidney.

**Figure 4:**
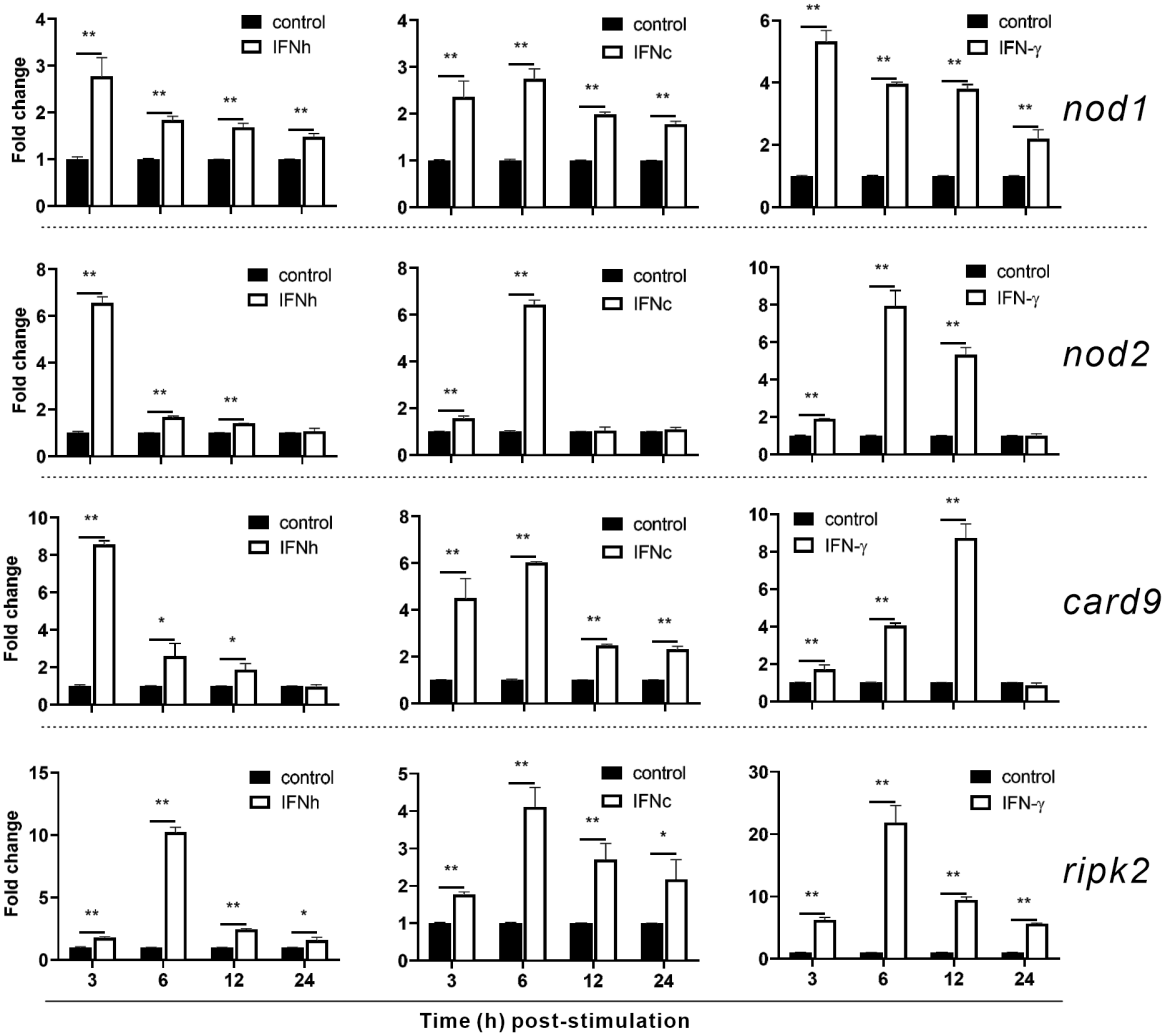
The induction of *nod1*, *nod2*, *card9* and *ripk2* genes by recombinant type I and type II IFNs. HEK293T cells were transfected with pcDNA3.1 empty vector (control), pcDNA3.1-IFNh, pcDNA3.1-IFNc or pcDNA3.1-IFN-γ plasmids to generate recombinant protein of IFNh, IFNc or IFN-γ, respectively. MFF-1 cells (5.0 × 10^5^) were incubated with the recombinant proteins of IFNh (final concentration = 107.5 ng/mL), IFNc (266.67 ng/mL), and IFNγ (57.92 ng/mL), respectively, for qRT-PCR assays. Gene expression was normalized against β-actin. Data were expressed as mean ± SE, with * indicating *P* < 0.05, **P < 0.01.

To investigate whether *nod1*, *nod2*, *ripk2* and *card9* can be induced by PAMPs and virus, the expression of the four genes was detected in poly(I:C)-treated and IHSV-infected MFF-1 cells, respectively. Compared to the control, poly(I:C) stimulation and IHSV infection all resulted in significant increase in the expression of *ripk2* and *card9* genes at different time points, and the induced expression of *nod1* and *nod2* genes was also observed (**Figures 3B, C**, **Supplementary Figure 3**). For further investigating whether *nod1*, *nod2*, *ripk2* and *card9* genes could be upregulated by IFNs, the expression of the four genes was detected in MFF-1 cells incubated with recombinant type I (IFNh and IFNc) and type II (IFN-γ) IFN proteins, which have been confirmed to induce ISGs, such as *mx, pkr* and *viperin* (**Supplementary Figures 4A-E**). It was observed that all the IFNs significantly induced the expression of *nod1*, *nod2*, *ripk2* and *card9* genes at 3 and 6 hours post-stimulation (**Figure 4**).

### 3.3 Identification of *nod1* and *nod2* as ISGs

**Figure 5:**
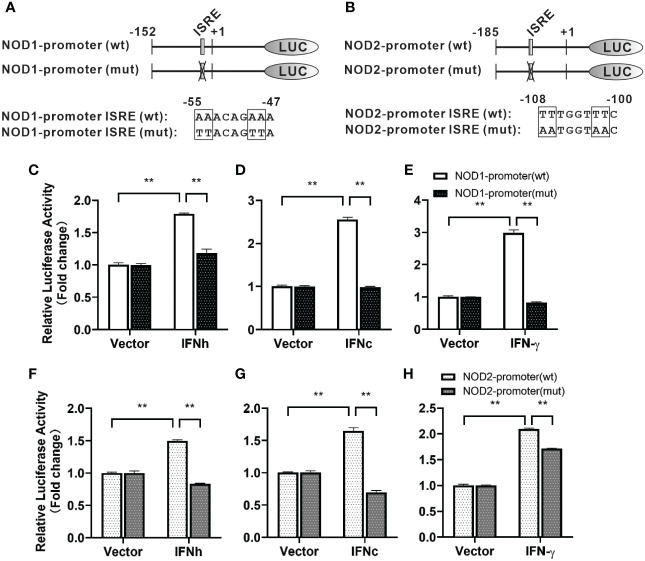
The induction of *nod1* and *nod2* genes by recombinant IFNs through ISRE. NOD1 **(A)** and NOD2 **(B)** luciferase reporter vectors with wild-type (wt) and mutant (mut) ISRE sites were indicated by schematic diagram. Activation of NOD1 **(C–E)** and NOD2 **(F–H)** luciferase reporter plasmids was mediated by recombinant IFNs. The promoter reporter vectors, including the ISRE-wt and ISRE-mut plasmids of NOD1/NOD2 were transfected into MFF-1 cells, which were then incubated with or without (control) the recombinant IFN proteins for 24 h to determine the luciferase activity. Data were expressed as mean ± SE, with ** indicating P < 0.01.

For understanding inducible regulatory elements of *nod1* and *nod2* genes, 5’-flanking sequences of the proximal promoter were identified. A potential ISRE site was found in the proximal promoters of both *nod1* and *nod2* genes, which were cloned to construct luciferase reporter plasmids with wildtype (wt) or mutant (mut) ISRE site (**Figures 5A, B**). The recombinant IFN proteins (IFNh, IFNc and IFN-γ) significantly activated ISRE-wt promoter plasmids of both *nod1* and *nod2* genes, but not the ISRE-mut plasmids (**Figures 5C–H**).

### 3.4 Interaction of NOD1, NOD2, RIPK2 and CARD9

**Figure 6:**
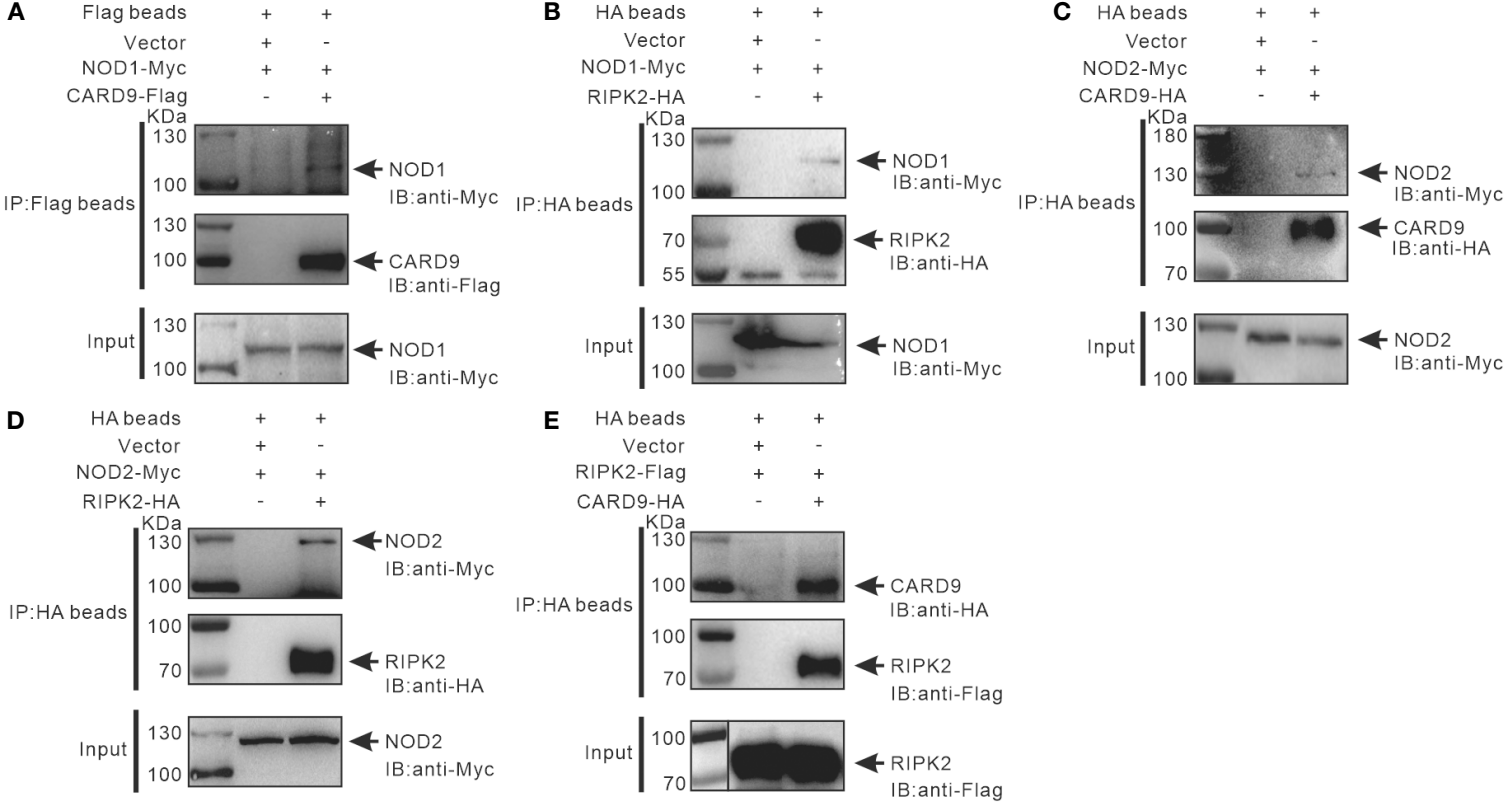
Interaction of NOD1-CARD9 **(A)**, NOD2-RIPK2 **(B)**, NOD2-CARD9 **(C)**, NOD2-RIPK2 **(D)** and RIPK2-CARD9 **(E)**. HEK-293T cells (about 5 × 10^6^ cells/well) were co-transfected with plasmids including NOD1-Myc, NOD2-Myc, CARD9-HA, RIPK2-HA, RIPK2-Flag, or empty vectors, for 48 hours to perform Co-IP using anti-Flag or -HA conjugated magnetic beads. The protein samples were then analyzed by Western blotting with anti-Myc, -Flag and -HA antibodies.

**Figure 7:**
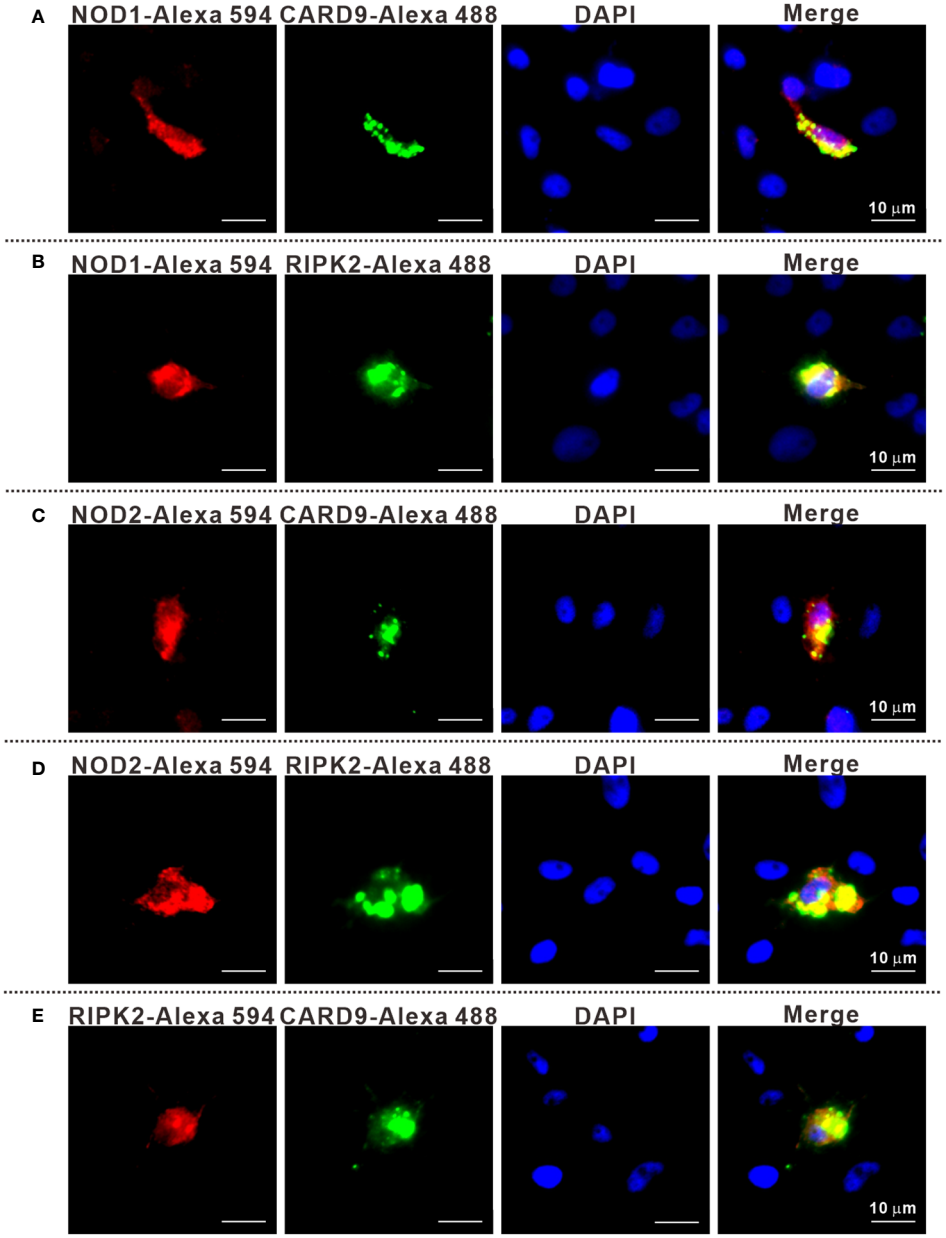
The subcellular colocalization of NOD1-CARD9 **(A)**, NOD2-RIPK2 **(B)**, NOD2-CARD9 **(C)**, NOD2-RIPK2 **(D)** and RIPK2-CARD9 **(E)**. MFF-1 cells were co-transfected with expression plasmids including NOD1-Myc, NOD2-Myc, CARD9-HA, RIPK2-HA, RIPK2-Flag, or empty vectors, for 24 hours. The cells were fixed by using 4% paraformaldehyde and were permeated by using 0.2% Triton X-100 to perform immunofluorescence assays with the fluorescein-labelled secondary antibodies. The cells were observed under a Leica confocal microscope (Scale bars = 10 μm).

It has been reported that RIPK2 and CARD9 as adaptors play a role in NLR-mediated immune signaling (11, 29, 30). In order to investigate whether RIPK2 and CARD9 are involved in NOD1/NOD2 signal complex, eukaryotic expression plasmids of the four genes were constructed. Co-IP assay was performed to determine the potential interaction of NOD1, NOD2, RIPK2 and CARD9 through pair-wise co-transfection of expression plasmids with tagged fusion protein, respectively. It was revealed that NOD1-Myc was associated with CARD9-Flag and RIPK2-HA (**Figures 6A, B**), and anti-HA affinity magnetic-immunoprecipitated CARD9 and RIPK2 were associated with NOD2-Myc (**Figures 6C, D**). Interestingly, CARD9-HA and RIPK2-Flag showed activity of interaction (**Figure 6E**). In addition, subcellular colocalization analysis also revealed the interaction of tagged NOD1, NOD2, RIPK2 and CARD9 through immunofluorescence. Colocalizations were observed for NOD1-CARD9 (**Figure 7A**), NOD1-RIPK2 (**Figure 7B**), NOD2-CARD9 (**Figure 7C**), NOD2-RIPK2 (**Figure 7D**), and CARD9-RIPK2 (**Figure 7E**).

### 3.5 Immune signaling of NOD1, NOD2, RIPK2 and CARD9

**Figure 8:**
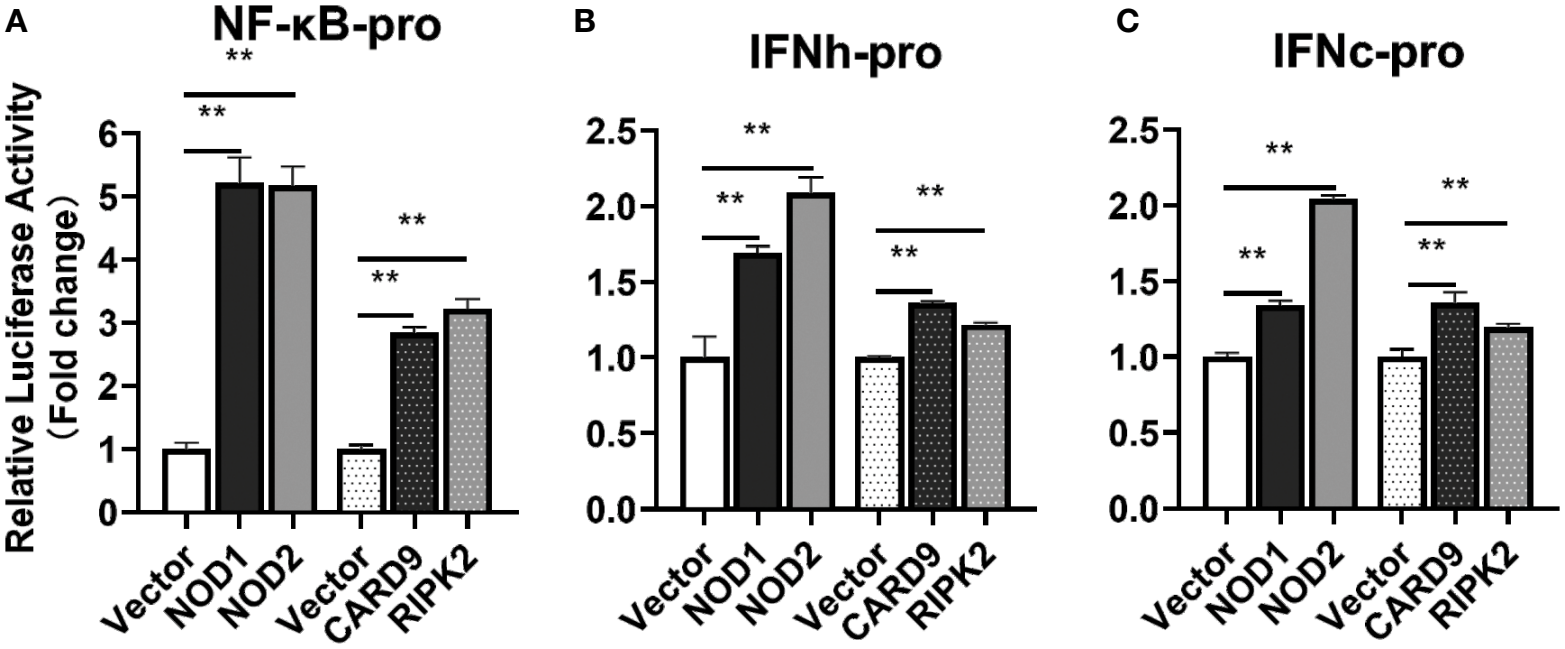
The activation of NF-κB **(A)**, IFNh **(B)** and IFNc **(C)** signaling by NOD1, NOD2, CARD9 and RIPK2. The promoter reporters (NF-κB, IFNh and IFNc) and expression plasmids (NOD1, NOD2, CARD9 and RIPK2) were respectively co-transfected into MFF-1 cells to determine the luciferase activity. Data were expressed as mean ± SE, with ** indicating P < 0.01.

**Figure 9:**
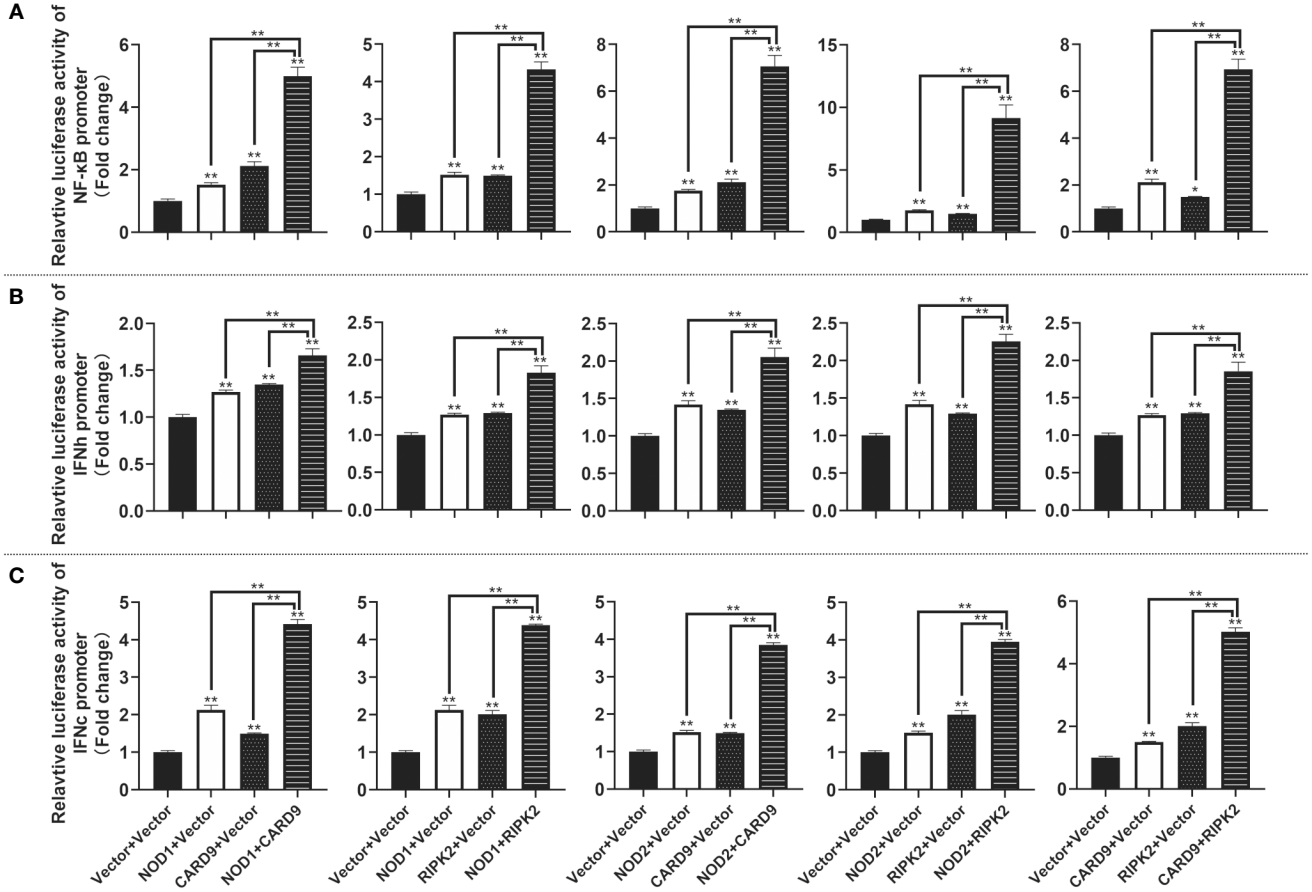
The activation of NF-κB **(A)**, IFNh **(B)** and IFNc **(C)** signaling by co-expression of NOD1, NOD2, CARD9 and RIPK2. The promoter reporters (NF-κB, IFNh and IFNc) and co-expression plasmids (empty vector, NOD1, NOD2, CARD9 and RIPK2) were respectively co-transfected into MFF-1 cells to determine the luciferase activity. Data were expressed as mean ± SE, with * indicating *P* < 0.05, **P < 0.01.

**Figure 10:**
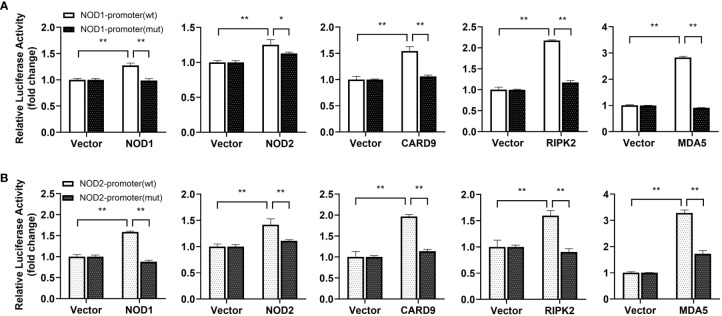
Promoter activation of *nod1* **(A)** and *nod2* **(B)** mediated by NOD1, NOD2, CARD9, RIPK2 and MDA5. The promoter reporters for NOD1 and NOD2 and expression plasmids (NOD1, NOD2, CARD9, RIPK2 and MDA5) were separately co-transfected into MFF-1 cells to determine the luciferase activity. Data were expressed as mean ± SE, with *, **, indicating *P* < 0.05, *P* < 0.01.

For understanding the immune signal mediated by NOD1, NOD2, RIPK2 and CARD9, promoter reporter plasmids of NF-κB, IFNh, IFNc, NOD1 and NOD2 were co-transfected with expression plasmids of NOD1, NOD2, RIPK2, CARD9 and MDA5 in MFF-1 cells, respectively. As shown in **Figures 8A–C**, the overexpression of NOD1, NOD2, RIPK2 and CARD9 provoked significantly the activity of luciferase reporter plasmids of NF-κB, IFNh and IFNc in MFF-1 cells, respectively. Furthermore, it was observed that the co-expression of these signal molecules, including NOD1 + CARD9, NOD1 + RIPK2, NOD2 + CARD9, NOD2 + RIPK2 and CARD9 + RIPK2, induced a significantly higher level of NF-κB, IFNh and IFNc promoter activity compared with the overexpression of these signal molecules alone, respectively (**Figures 9A–C**). Interestingly, the overexpression of NOD1, NOD2, RIPK2, CARD9 and MDA5 provoked significantly the activity of *nod1* and *nod2* promoters with wildtype ISRE, respectively, but not the promoters with mutant ISRE (**Figures 10A, B**).

## 4 Discussion

**Figure 11:**
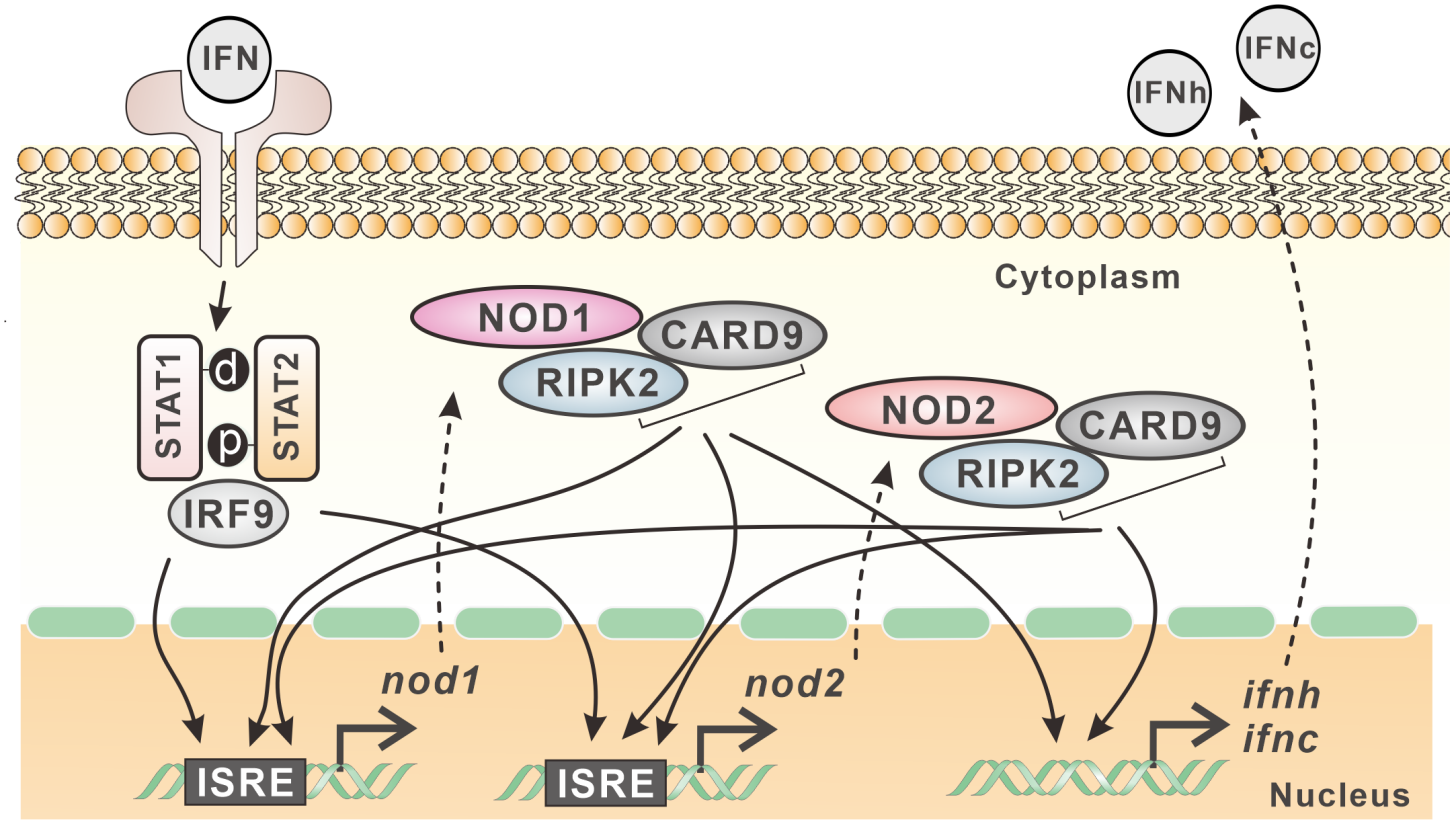
Expression regulation model of *nod1* and *nod2* in Chinese perch. NOD1, NOD2, CARD9 and RIPK2 show interactive relationship and contribute to the expression of type I IFNs. Furthermore, *nod1* and *nod2* genes contain ISRE in their promoters, and can be induced by type I IFNs, which in turn can be upregulated also by CARD9, RIPK2, and even by NOD1/NOD2 themselves.

In mammals, NOD1 and NOD2 mediated signaling is involved in antimicrobial immunity (9). However, the transcription and signaling complex of NOD1 and NOD2 are quite unclear in teleost fish. In the present study, with the cloning of *card9* and *ripk2* genes, the interaction of NOD1 and NOD2 with CARD9 and RIPK2 was revealed through colocalization and Co-IP. Interestingly, these four genes were found to be upregulated by type I and type II IFNs, i.e. IFNh and IFNc, and IFN-γ in Chinese perch. An ISRE was identified in the proximal promoter of *nod1* and *nod2* genes, and the overexpression of NOD1, NOD2, RIPK2 and CARD9 all resulted in the increase in reporter activity of NF-κB, and IFNh and IFNc, revealing the antiviral signaling of NOD1 and NOD2 in the perciform Chinese perch (**Figure 11**).

In response to viral infection, IFN signaling leads to the induction of a large group of genes called ISGs, some of which are responsible for interfering with viral replication and providing protection for host cells (2). A mass of ISGs with antiviral activity have been discovered in mammals, while in fish, their number is comparatively limited (31, 32). The proximal promoter of typical ISGs may contain specific DNA elements, i.e. ISRE, which can respond to IFN signaling by binding with a family of transcription factors, such as IFN stimulated gene factor 3 (ISGF3) (2). It has been reported that active ISRE sites are present in promoter region of ISGs in fish, such as MX, IFP35 and NMI, which shows conservative transcriptional mechanism in response to the stimulation of IFNs when compared with mammals (27, 33). Interestingly, some PRRs have been identified as ISGs, such as cyclic GMP-AMP synthase (cGAS), RIG-I, MDA5, and dsRNA-dependent protein kinase (PKR) (34). In fact, these ISGs can also be induced by synthetic dsRNA (poly I:C) and viral infection in addition to IFN stimulation (31). However, NOD1 and NOD2 are important members of NLR family, and their transcription mechanism is still unclear. In this study, the significantly induced expression of *nod1* and *nod2* by type I (IFNh and IFNc) and type II (IFNγ) IFNs, and also by poly(I:C), and significant increase in promoter activity of *nod1* and *nod2* through ISRE as revealed by dual-luciferase reporter assay may indicate that fish NOD1 and NOD2 may represent ancient members of ISGs, as NOD1 and NOD2 are evolutionarily conserved PRRs in vertebrates.

In mammals, NOD1/NOD2 interacts with RIPK2, leading to the production of proinflammatory cytokines, to play roles in autophagy and anti-bacterial, -viral and -parasitic infections (9, 10, 35). In bony fish, the interaction between RIPK2 and NOD1/NOD2 has been reported in zebrafish, goldfish (*Carassius auratus*), and miiuy croaker (*Miichthys miiuy*) (36–38). Structurally, NOD1 and NOD2 all contain a nucleotide oligomerization NACHT, a LRR domain, and one CARD/two CARDs, which is/are critical site(s) to interact with the CARD of RIPK2 (9). In mammal, CARD9 binds to NOD2 through NACHT or CARD-NACHT linker region of NOD2, but not through CARD-CARD interaction (39). Structurally, the two adaptor proteins have conserved CARD domain and possess additional CC or KD domain (9, 11, 40, 41). It is obvious that an N-terminal CARD and a C-terminal CC domain, and an N-terminal KD and a C-terminal CARD domain are also found in CARD9 and RIPK2 in Chinese perch, respectively. The conserved domain composition, and conserved gene loci for CARD9 and RIPK2 in fish as in mammals may imply their similar functional characteristics in vertebrates.

As mentioned above, NOD1, NOD2, CARD9 and RIPK2 in Chinese perch possess conserved domains, which may provide the binding basis for the proteins. Expectedly, NOD1 and NOD2 were associated with RIPK2, and the interaction between CARD9 and NOD1/NOD2/RIPK2 was also observed through Co-IP and immunofluorescence assay, suggesting that teleost CARD9 and RIPK2 are likely to be the components of signal transduction complex of both NOD1 and NOD2, as reported in mammals.

It has been reported that mammal NOD1 and NOD2 can induce antiviral response via RIPK2-TRAF3-IRF3/IRF7-IFN pathway to suppress the replication of viruses, such as human cytomegalovirus (HCMV), respiratory syncytial virus (RSV), or influenza A virus (IAV) (29, 42–45). To date, NOD1 has been demonstrated to be able to mediate RLR-associated antiviral signaling in bony fish, and this is accomplished by binding viral RNA and regulating the interaction between MDA5 and MAVS, thereby promoting antiviral signaling (20). NOD2 and RIPK2 can also activate NF-κB and type I IFN promoters in fish, inducing significant antiviral defense against SVCV infection (46). In this study, the overexpression of NOD1, NOD2, and RIPK2 significantly induced the activity of NF-κB, IFNh and IFNc promoter reporters, indicating the conserved signal pathway mediated by NOD1 and NOD2 in teleost. Interestingly, mammal CARD9 was reported to be involved in the process of RIG-I mediated NF-κB activation against RNA viruses (47). In this study, CARD9 was found to be interactive with all the three key molecules, NOD1, NOD2 and RIPK2, implying that teleost CARD9 is involved in NOD1 and NOD2 mediated signaling, and CARD9 can also induce the promoter activity of NF-κB and type I IFNs. Furthermore, NOD1 and NOD2 as the critical PRRs were induced by MDA5, CARD9, RIPK2, IFNs, as well as NOD1/NOD2 themselves through ISRE in Chinese perch, suggesting the positive-feedback regulation pathway of NOD1/NOD2-IFNs in teleost.

In summary, with the identification of CARD9 and RIPK2 in Chinese perch, the interactive relationship between NOD1/NOD2 and CARD9/RIPK2 was demonstrated, contributing to the expression of type I IFNs. Furthermore, *nod1* and *nod2* genes contain ISRE in their promoters, and can be induced by type I IFNs, which in turn can be upregulated also by MDA5, CARD9, RIPK2, and even by NOD1/NOD2 themselves.

## Data availability statement

The datasets presented in this study can be found in online repositories. The names of the repository/repositories and accession number(s) can be found in the article/**Supplementary Material**.

## Ethics statement

The animal studies were approved by Institute of Hydrobiology, Chinese Academy of Sciences. The studies were conducted in accordance with the local legislation and institutional requirements. Written informed consent was obtained from the owners for the participation of their animals in this study.

## Author contributions

XYP: Conceptualization, Investigation, Writing – original draft, Writing – review & editing, Data curation, Formal Analysis. KLW: Investigation, Writing – original draft, Resources, Data curation. LL: Data curation, Investigation, Writing – original draft. BL: Data curation, Writing – original draft, Methodology. XYW: Writing – original draft, Investigation. ZWZ: Investigation, Writing – original draft, Resources. NL: Resources, Writing – original draft, Data curation. LHL: Resources, Writing – original draft, Project administration. PN: Project administration, Resources, Writing – original draft, Conceptualization, Investigation, Writing – review & editing, Validation, Funding acquisition, Supervision. SNC: Data curation, Investigation, Writing – original draft, Conceptualization, Formal Analysis, Validation, Writing – review & editing.
