# Supplementary material for: Transcription of NOD1 and NOD2 and their interaction with CARD9 and RIPK2 in IFN signaling in a perciform fish, the Chinese perch, Siniperca chuatsi
Source: Front Immunol. 2024 Apr 23;15:1374368. doi: 10.3389/fimmu.2024.1374368 (PMC11074466; doi:10.3389/fimmu.2024.1374368)
Supplement: Supplementary file 1 [file DataSheet_1.pdf]

Supplementary Material with one Supplementary Table and four Supplementary Figures

Supplementary Table 1. PCR primers used in this study.

Primer	Sequence 5'-3'	Usage
NOD1F1	ACGCAACACTATGAGGTGACT	Gene cloning
NOD1R1	GTCACTAATGGCCTTCAGATG	
NOD2F1	TCAGTACATGTTGGATGTCCC	
NOD2R1	AGTGGCTAAGGACTGTATAT	
CARD9F1	ACTCAGAGTGATGGAGGGAGT	
CARD9R1	ATTCAGCTGCTGACGGTGTG	
RIPK2F1	CACTGGCTGACTTAGTGGATG	
RIPK2R1	CAATCCAGATTATAGACTGTG	
MDA5F1	GTGTTACCGGTTAGCTGGGTC	
MDA5R1	CACATAAACACTGATCTCTG	
Qscβ-actin-F	GAGAGGGAAATCGTGCGTGA	qRT-PCR
Qscβ-actin-R	CATACCGAGGAAGGAAGGCTG	
QscNOD1-F	ACTGTGCGTCAATCAGCTGTCT	
QscNOD1-R	ACCGATCTTGACCATTCGCAGC	
QscNOD2-F	GCTCTGTTGTGGCTTAGCCT	
QscNOD2-R	GAATCAGACATTCCACTCCTG	
QscCARD9-F	TGACCTCCAGAAGTCGACCA	
QscCARD9-R	CGGAGTCGGTGATGTCACTG	
QscMx-F	GCCCAAAGTAGCTTCACTGGA	
QscMx-R	CCGTTTGTCTTCCTCCTCATC	
QscViperin-F	ACTGTCGAGCAGGCAGGAAGGA	
QscViperin-R	CACCACTGCAGCTTCATGTTGGC	
QscPKR-F	ATCCACAGAGACCTGAAGCCTG	
QscPKR-R	GCTCAGGAGCCATGTAAGATGG	
Myc-NOD1-F	CCGGAATTCATGGGTCAAAAAGAAGAAGCC	plasmid construction
Myc-NOD1-R	GCACTCAAAGAGATATGGGGTACCCCG	
Myc-NOD2-F	CCGGAATTCATGTTTGTCCAGGAGCTTGTG	
Myc-NOD2-R	CGCGAATCAAGACTGATCTTCGGTACCCCG	
Flag-CARD9-F	AAGGAAAAAAGCGGCCGCATGGAGGGAGTGTGTGA	
Flag-CARD9-R	GCAGTGACATCACCGACTCCGACTCTAGACTAG	
HA-CARD9-F	CCGGAATTCATGGAGGGAGTGTGTGAGGACG	
HA-CARD9-R	GACATCACCGACTCCGACTACCCATACGACGTCCCAGACTACGCTTAGTCTAGACTAG	
Flag-RIPK2-F	AAGGAAAAAAGCGGCCGCATGATAAATCCTCGGAG	
Flag-RIPK2-R	TCCTACAATATCCCCAGGAACATGTCTAGACTAG	
HA-RIPK2-F	CCGGAATTCATGATAAATCCTCGGAGAC	
HA-RIPK2-R	ACAATATCCCCAGGAACATGTACCCATACGACGTCCCAGACTACGCTTAGGGTACCCCG	
HA-MDA5-F	AAGGAAAAAAGCGGCCGCATGACATCGGACAACGA	
HA-MDA5-R	AGTGACAACTACGTACCCATACGACGTCCCAGACTACGCTTAGGGTACCCCG	

Supplementary Figures

Supplementary Figure 1

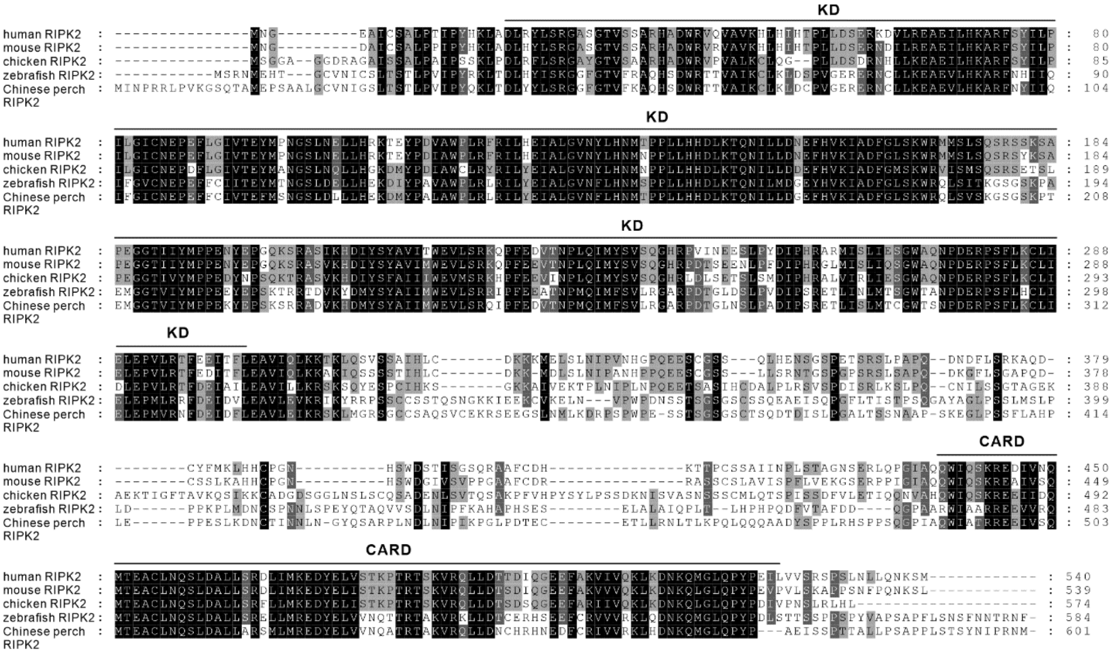

Supplementary Figure 1. Multiple alignment of RIPK2 amino acid sequences. Identical and similar amino acids are indicated with black and grey shadow, respectively.

Supplementary Figure 2

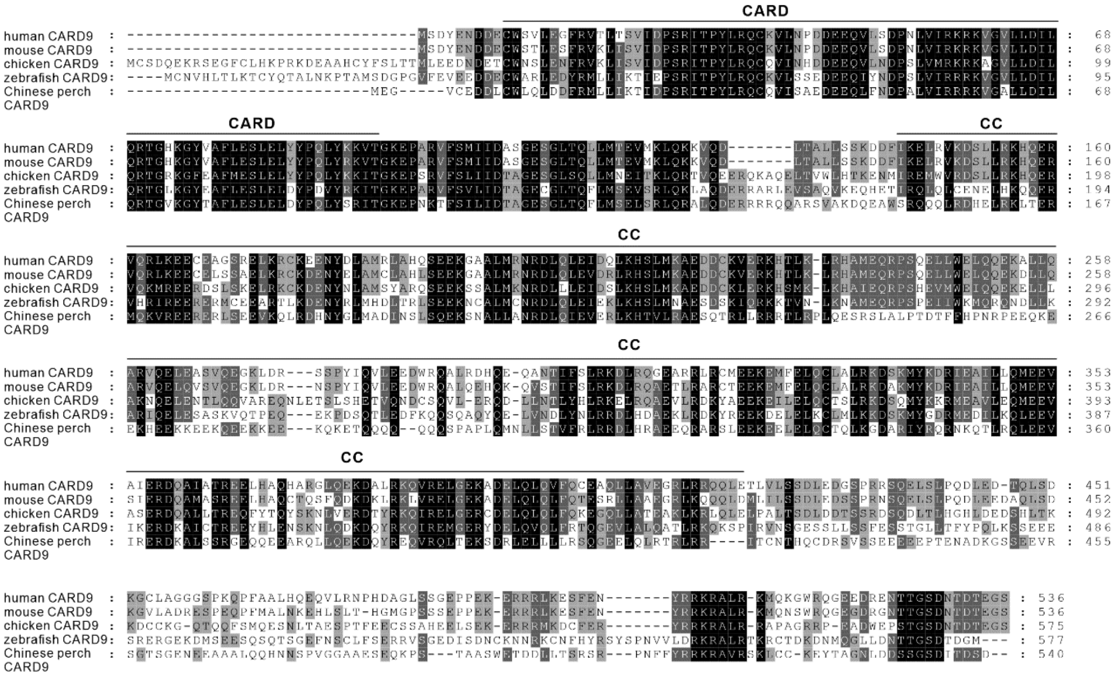

Supplementary Figure 2. Multiple alignment of CARD9 amino acid sequences. Identical and similar amino acids are indicated with black and grey shadow, respectively.

Supplementary Figure 3

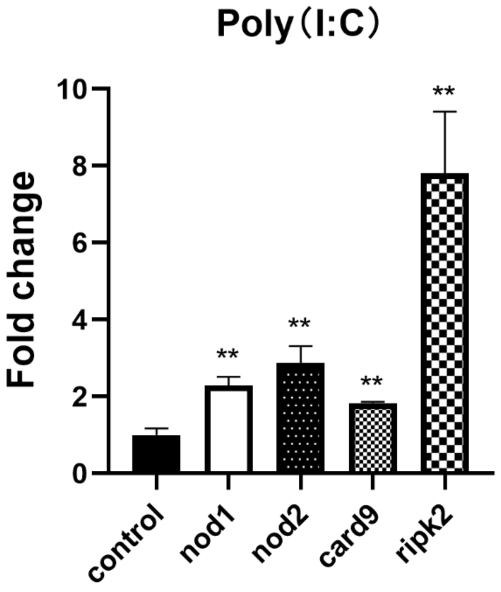

Supplementary Figure 3. Induction of *nod1, nod2, card9* and *ripk2* genes in MFF-1 cell line by poly(I:C). MFF-1 cells (5.0 × 10^5) were transfected with of a final concentration of 8.33 μg/mL poly(I:C) and were collected at 12 h post-stimulation. Untreated cells were set as control. Gene expression was determined by qRT-PCR and was normalized against β-actin. Data were expressed as mean ± SE, with * indicating $P < 0.05$, ** $P < 0.01$.

Supplementary Figure 4

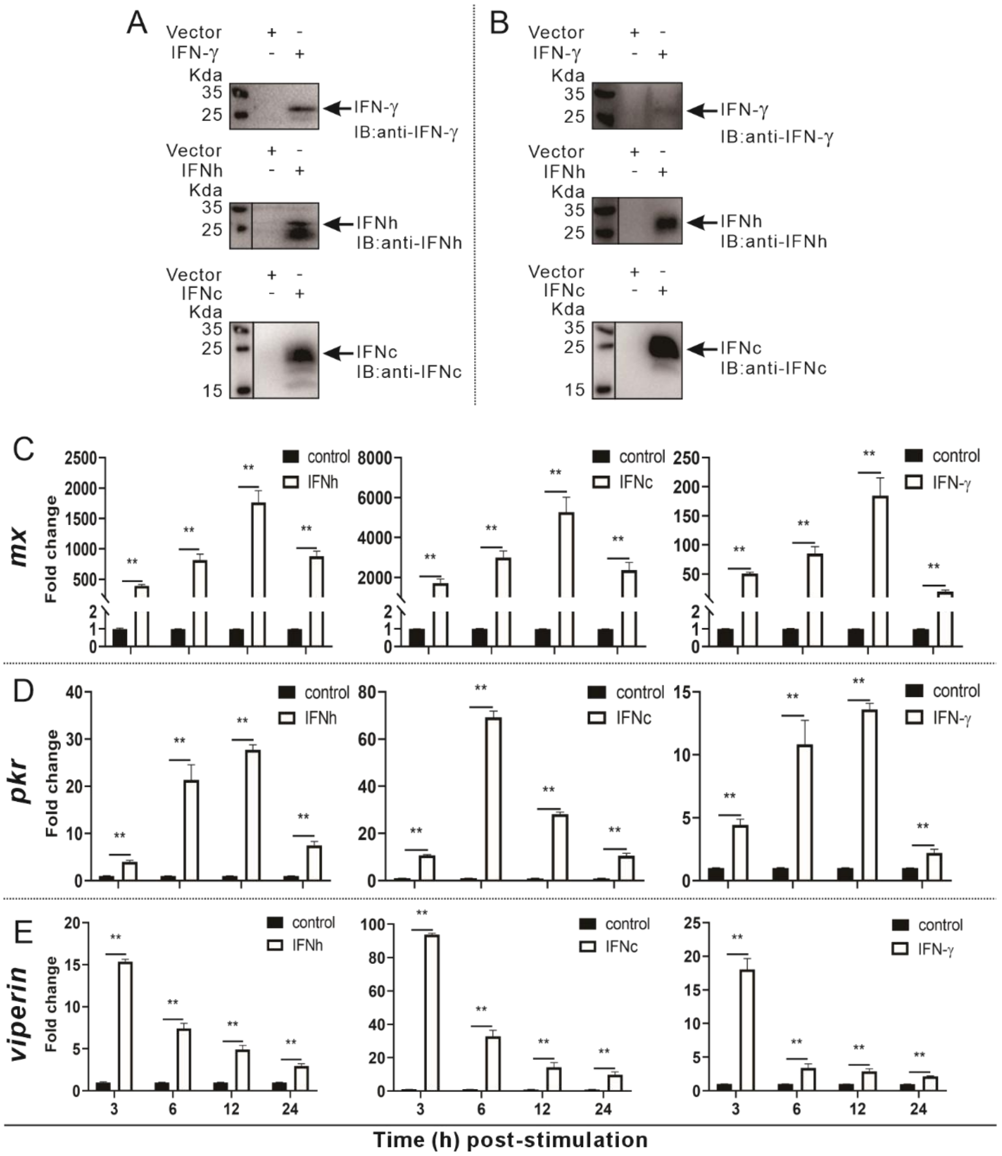

Supplementary Figure 4. The recombinant type I and type II IFN proteins. HEK293T cells were transfected with pcDNA3.1 empty vector (control), pcDNA3.1-IFNh, pcDNA3.1-IFNc or pcDNA3.1-IFN-γ plasmids to generate recombinant proteins of IFNh, IFNc or IFN-γ, which were detected by using Western blotting with the IFN-specific antibodies in the precipitation (A) and supernatant media (B) of plasmid-transfected HEK293T cells, respectively. MFF-1 cells (5.0×10^5) were incubated with the control medium and the supernatant with recombinant protein of IFNh, IFNc or IFNγ, respectively, for qRT-PCR assays. Gene expression of ISGs (*mx, pkr* and *viperin*) was normalized against β-actin. Data were expressed as mean ± SE, with * indicating $P < 0.05$, ** $P < 0.01$.
